# Healthy publics: enabling cultures and environments for health

**DOI:** 10.1057/s41599-018-0113-9

**Published:** 2018-05-15

**Authors:** Stephen Hinchliffe, Mark A. Jackson, Katrina Wyatt, Anne E. Barlow, Manuela Barreto, Linda Clare, Michael H. Depledge, Robin Durie, Lora E. Fleming, Nick Groom, Karyn Morrissey, Laura Salisbury, Felicity Thomas

**Affiliations:** 1 Department of Geography, College of Life and Environmental Sciences, University of Exeter, Exeter, UK; 2 Department of History, College of Humanities, University of Exeter, Exeter, UK; 3 Child Health and Health Complexity, Institute for Health Research, University of Exeter Medical School, Exeter, UK; 4 University of Exeter Law School, Exeter, UK; 5 Department of Psychology, University of Exeter, Exeter, UK; 6 Lisbon University Institute (CIS/ISCTE-IUL), Lisbon, Portugal; 7 Centre for Research in Ageing and Cognitive Health, University of Exeter, Exeter, UK; 8 European Centre for the Environment and Human Health, University of Exeter Medical School, Truro, UK; 9 Department of Politics and International relations, University of Exeter, Exeter, UK; 10 Department of English, University of Exeter, Exeter, UK; 11 Department of English and Film, University of Exeter, Exeter, UK; 12 University of Exeter Medical School, Exeter, UK; 13 College of Humanities, University of Exeter, Exeter, UK

## Abstract

Despite extraordinary advances in biomedicine and associated gains in human health and well-being, a growing number of health and well-being related challenges have remained or emerged in recent years. These challenges are often ‘more than biomedical’ in complexion, being social, cultural and environmental in terms of their key drivers and determinants, and underline the necessity of a concerted policy focus on generating healthy societies. Despite the apparent agreement on this diagnosis, the means to produce change are seldom clear, even when the turn to health and well-being requires sizable shifts in our understandings of public health and research practices. This paper sets out a platform from which research approaches, methods and translational pathways for enabling health and well-being can be built. The term ‘healthy publics’ allows us to shift the focus of public health away from ‘the public’ or individuals as targets for intervention, and away from the view that culture acts as a barrier to efficient biomedical intervention, towards a greater recognition of the public struggles that are involved in raising health issues, questioning what counts as healthy and unhealthy and assembling the evidence and experience to change practices and outcomes. Creating the conditions for health and well-being, we argue, requires an engaged research process in which public experiments in building and repairing social and material relations are staged and sustained even if, and especially when, the fates of those publics remain fragile and buffeted by competing and often more powerful public formations.

## Introduction

Despite extraordinary advances in biomedicine and associated gains in human health prospects, a growing number of health and well-being related challenges remain or have emerged in recent years. These challenges are often more than biomedical in complexion, being social, cultural and environmental in terms of their key drivers and determinants. There are numerous examples: mental health is linked to lifestyles, as well as to the environments and economies in which people live; antibacterial resistance is related to social demand for, delivery and use of available medicines; the needs of ageing populations, the causes and health impacts of accelerated climate change, persistent local and international inequalities in health, and the damaging health effects of poverty, isolation and loneliness-all require an appreciation of complex social, cultural and environmental processes in order to create and sustain conditions for health and well-being.

Tackling these and other pressing national and international health and environmental issues requires commitment not only to reducing the burden of disease, but also to strengthening people-centred health systems (World Health Organisation, 2012) and public health capacity (Davies et al., 2014; Hanlon et al., 2011), recognising the cultural contexts of health (Greenhalgh, 2016; Kagawa Singer et al., 2016; Napier et al., 2014), focusing on the quality of our social and ecological relations (Whitmee et al., 2015b), and investing in health through a life-course approach (The Academy of Medical Sciences, 2016). An emerging if under-specified consensus across these documents suggests that this turn to the conditions of possibility for health necessitates establishing novel interdisciplinary research partnerships and building and sustaining cross-sectoral collaborations with various communities and stakeholders (World Health Organisation, 2012, 2015b).

Adding substance and detail to these calls for new partnerships and practices requires a radical re-assessment of our approaches to health and well-being, to forms of working and the evidence base on which health policies are developed. In this paper we set out a conceptual platform from which innovative research methods and translational pathways for enabling health and well-being can emerge. After critically reviewing recent arguments for greater awareness of the cultural and environmental determinants of health and for participatory approaches to research, we introduce the notion of healthy publics. This is a term we use to describe dynamic collectives of people, ideas and environments that can enable health and well-being. These fragile collectives may be distributed across many areas of expertise, be geographically or spatially diverse, draw on a range of matters and materials that evidence their claims to health, and, importantly, can use their array of experiences and material relationships to question received or established approaches to health. We illustrate our argument with a number of cases, drawn from our own and others’ recent research and presented here as a means to exemplify, rather than exhaust, the arguments for, and instances of, healthy publics.

We argue that by enriching understandings of cultural practice and generating innovative approaches to health and well-being, healthy publics can shift the focus of public health away from populations and individuals as passive targets for policy, and away from the view that culture acts as a barrier to efficient biomedical intervention. Instead, healthy publics underline the importance of social, cultural and environmental movements and relations in providing the conditions for healthy outcomes across the life course.

## Problematising the cultural turn in public and planetary health

Recent debates about public health have highlighted the need for a shift in emphasis from a focus on the determinants of disease and towards approaches that foster the conditions for health, well-being and sustainable, healthy, environments. For example, reports from international and national organisations such as the World Health Organisation, the Rockefeller Foundation-*Lancet* Commission, the Academy of Medical Sciences, the Campaign for Social Science, and the Arts and Humanities Research Council in the UK have all drawn attention to the limited capacity of biomedical research alone to address ‘increasingly diverse and complex [health] issues that transcend disciplinary, sectoral and geographical boundaries’ (The Academy of Medical Sciences, 2016, p 5, see also Campaign for Social Science, 2017; World Health Organisation, 2012). In so doing, they have highlighted the need to move away from individually-focused behavioural interventions to a relational approach that creates conditions for health. In spite of adopting slightly different perspectives on the recommended pathways to health and well-being, these arguments for innovative and sustainable methods of enabling human health and well-being, as well as the health of other species and the planet, share a number of characteristics that can inform an approach to healthy publics.

First, these approaches prioritise ‘culture’ as a key determinant of health. Of course, interest in the relationship between culture and health is not new; it was, for example, built into the formulation of transcultural psychiatry in the post-Second World War years and has been one of the key features of approaches to HIV/AIDS prevention and care since the 1980s (UNESCO, 2010). Recent reviews, however, have emphasised the need to reinvigorate interest in cultural, as well as social, contexts in order to address inequalities in health and well-being, and to integrate evidence drawn from studying such contexts into health policy and practice. The *Lancet* Commission on Culture and Health has made the provocative assertion that the ‘systematic neglect of culture’ constitutes the ‘single biggest barrier to advancement of the highest attainable standard of health worldwide’ (Napier et al., 2014). This has been endorsed elsewhere: in World Health Organisation (WHO) Europe’s commitment to foregrounding the cultural contexts of health as part of Health 2020 (World Health Organisation, 2015a; World Health Organisation, 2016); in the UK Chief Medical Officer’s arguments for a ‘cultural wave’ (or ‘fifth wave’) of public health (Davies et al., 2014; Hanlon et al., 2011); in references to culture as the ‘missing link’ in health research (Kagawa Singer et al., 2016); and in the growing interest in the interrelations between natural, built and work environments, cultural values, and health (Clark et al., 2014).

While this somewhat belated turn to culture is welcome, there is an inclination in some if not all of these pronouncements to present culture as a set of norms, properties or established ways of doing things. Culture in these documents generally corresponds to the UNESCO definition as ‘the set of distinctive spiritual, material, intellectual and emotional features of society or a social group, and that it encompasses, in addition to art and literature, lifestyles, ways of living together, value systems, traditions and beliefs’ (UNESCO, 2002). Although this formulation is constructive, bringing to the fore questions about material production, social relations and symbolic systems, it can lead to the tendency to treat culture as distinct from, and therefore a barrier to, the efficient application of scientific or biomedical knowledge. Similarly, and vitally, it neglects the incompleteness of culture, its dynamism, and the tendency for subgroups to challenge established practices, to innovate, and to borrow from other groups and cultures (Hall, 2016).

It is therefore important to emphasise that cultures are neither neat, nor homogeneous, nor do they operate as a ‘single integrated reality’ (Scheper–Hughes, 1984); rather, they are emergent, continually shaped and reshaped by power and inequality, and characterised by diversity, hybridity and exchange. These dynamic features of cultures are key to understanding why and how healthy publics operate within and through a set of continuous struggles over meaning and evidence.

Second, there is renewed emphasis on the significance of taking seriously the forms of evidence generated by methods that elicit the beliefs, practices, values, and social processes that can shape how health and well-being are understood and practised. These methods are often, though not always, qualitative, and are able to generate improved understandings of cultural practices. Like quantitative research, qualitative approaches can be judged not only on their rigour, but also on their ability to provide insights into the complex and multifactorial conditions for health and well-being. Indeed, proponents of a ‘fifth-wave of public health’ have argued for the need to integrate narratives of lived experience with quantitative measurements of biological processes in order to understand and address social inequalities in health (Hanlon et al., 2011). Collating and analysing evidence of embodied experience and embedded social practices, and assimilating them into health and social care policies, is not without problems. But as Greenhalgh has demonstrated in a recent WHO report, ‘appropriate and rigorous use of narrative methods’ (alongside quantitative epidemiological forms of evidence) offers policy-makers more robust support for a ‘values-based approach that is better able to incorporate (and respond appropriately to) diverse cultural contexts’ (Greenhalgh, 2016).

Research across the humanities and social sciences demonstrates how evidence generated by oral histories, through focus groups and ethnographic methods or by studies of cultural heritage, enables us to: clarify how health and well-being are defined, re-defined and experienced; open up opportunities for more marginalised voices to be heard; understand more clearly the relational and historical dimensions of health and illness; and integrate life-course perspectives into research, policy and practice (World Health Organisation, 2015c). Qualitative evidence can also help to improve the interpretation of statistical data. Studies drawing on the lived experiences of women in Romania, for example, indicate how the quantitatively documented low uptake of both HPV vaccination and screening for cervical cancer–and the resulting high level of mortality from this disease - is related to how women’s sexual and reproductive health and well-being have been represented and politicised in media and state discourses (Johnson et al., 1996; Rada, 2014; Todorova et al., 2009; Todorova et al., 2006). Similarly, analysis of historical and literary sources, as well as cross-cultural comparisons, can reveal the social and cultural complexities of dominant Western narratives of ageing; and challenge standardised, predominantly chronological, calibrations of the life course (Lock, 1993; Gullette, 1997, 2004). However, although historical and anthropological studies have usefully exposed the cultural, political and economic drivers of certain forms of ageism, they have too often neglected to engage with personal experiences, to contest normative notions of the family, or to acknowledge fully the limits of autonomy, with the result that they serve to reproduce the power relations that they seek to disrupt (See Box 1).

It is important to note that re-investments in personalised accounts and cultural conditions are being augmented through access to larger and larger data sets and the production of health-related data through mobile technologies. These relatively new opportunities for engaging people through critical approaches to volunteered health data made possible by wearable technology, apps and other e-technologies (Lupton, 2018) lie behind some of these more optimistic versions of a new public health. Nevertheless, challenges remain in terms of ethical and social forms of consent to the use of such data, recognising and addressing inequalities of access to wearable technologies, interpreting the resulting forms of evidence, and determining how they might contribute to understandings of health and well-being.

The recognition of the power of evidence that has either previously been regarded as ‘lacking’ the attributes of medical sciences or offers new ways of constituting the (digital) body is of course welcome. Yet, as we suggest through the notion of healthy publics, these data and the methods through which they are generated will undoubtedly compete in what are crowded and noisy fields that remain riven with uneven power relations and existing epistemic commitments. How these forms of working fare in this public domain of an information- rich society with a surfeit of data and knowledge on health is a matter for careful future investigation.

Our third point is that if culture and personal accounts are central to enabling health, it becomes imperative that people with various forms of direct and/or relevant experience of a health-related issue can be active partners in the research process and can contribute from the beginning to identifying, prioritising, designing, conducting and disseminating more participatory, action-based research. Such an approach allows health-creating policies and practices to be aligned with the values, needs and expectations of diverse groups within society. Along these lines, a recent report has suggested that meaningful public engagement is a necessary condition for health creation in the UK National Health Service: enabling a shift away from the ‘“factory” model of care and repair, with limited engagement with the wider community’ (NHS England, n.d.), towards something that harnesses existing cultural resources for creativity and potentiality for health and well-being. We would only add that engagement is also a vital means of highlighting and suggesting ways of mitigating material and institutional barriers to the realisation of these opportunities.

This need to engage with civil society and community organisations to develop ‘novel coalitions’ for health and well-being is equally prominent in calls for a shift to Planetary Health – a term used to emphasise the interrelations of human health and the ecological and geophysical processes on which it depends. This expanded sense of health requires a wide public mandate, including corporate bodies and wealthy consumers, but also it will necessarily involve ‘the participation, commitment, and ownership of those most affected by threats to health from the degradation of the biosphere,’ including ‘indigenous communities, the poorest billion living in the most marginal environments of the developing world, the rural poor, and the urban poor in the sprawling cities’ (Whitmee et al. 2015a: 2014, see also Wellcome Trust, n.d.).

While these call to arms are welcome, there remains a general lack of specificity regarding the means and ends, and the inevitable constraints, of these participatory approaches. Certainly, we can learn from the ways in which traditional public participation in research and policy formation have strengthened culturally-informed understandings of well-being and begun to shape policy (Oxfam, 2015).^1^ But these coalitions for health will be impeded by, as well as have to contend with, the very inequalities and uneven power relations that made them necessary in the first place. How publics manage their internal relations as well as continuously present and engage themselves in a crowded public sphere requires more consideration. These collectives will take serious and intense experimentation and work.

If we are to incorporate cultural, environmental, relational and participatory approaches into health research and policy that adequately address current and future health challenges, we need to develop innovative partnerships that integrate ‘aspects of natural, social and health sciences, alongside the arts and humanities’ (The Academy of Medical Sciences, 2016). At the same time, enabling health and well-being across the life course requires us to prioritise the creation of collective solutions that involve ‘all sectors of government, academia, civil society, the private sector and the media’ (World Health Organisation, 2015c).

This, we recognise, requires active participation that involves communities, researchers, and others co-generating meaningful research questions and study designs, debating and contesting what counts as evidence for healthy outcomes, and working across existing disciplinary and institutional boundaries. These are characteristics of a transformative and transdisciplinary approach to culturally informed and engaged research. As we have suggested, all of this requires further thought, experimentation and specification. In what follows, we set out a framework for that endeavour. In particular, we explore the notion of healthy publics, and provide indicative examples of how transdisciplinary research that engages with diverse groupings from the outset can enable and sustain conditions for health and well-being.

## Healthy publics

It is now well established that neglect of the social, cultural, historical, and environmental contexts of health can result in poor outcomes. Nevertheless, questions remain about *how* best to bring these various components together. How can those with expertise and experience in these various fields collaborate in novel ways to enable health? More specifically, what does it take to mobilise and sustain transdisciplinary groupings that can redefine and improve health and well-being?

Given our interpretation of a healthy public as a dynamic collective of people, ideas and environments that enable health and well-being, we take it as read in the first instance that this grouping involves those with lived experience of, expertise in, or a history of exclusion from, health and well-being matters. This collective will need to be more diverse than in traditional forms of research and practice, involving lay and professional expertise working at a particular site, or across a number of locations, even internationally. It goes without saying, we hope, that constituting this public will itself involve ongoing but creative struggles over agenda, meanings, and forms of working.

Second, the resulting assembly of expertise and experiences may well generate new insights and knowledge that challenge existing practices, knowledge and norms concerning health and well-being. This potential of emerging collectives to raise new questions, to challenge received wisdom on what is healthy and unhealthy, is a key component of the openness of healthy publics.

Third, in querying norms and generating new knowledge, these collectives can divide as well as bring together - and may confront - other collectives with different interests and expertise. Indeed, the key division in healthy publics is not between lay and expert knowledge, but within and between different groupings of experts, civil society, business interests and so on, each with varying levels of resource, social power and access to, as well as preferences for, different kinds of evidence (Irwin and Michael, 2003). Healthy publics are therefore not only public in terms of their knowledge generation, they are also players in an often crowded and contested public sphere of health claims and counter claims.

To be clear, this sense of healthy publics as a generative and contested collective is quite different to more established senses of public health. In traditional deficit-led approaches, *the* public is understood to be already out there, a population waiting (passively) to be informed or incentivised about an issue. It is a target and the aim is to produce an ‘informational citizen’ through judicious use of behavioural economics, public understanding of science, and arts-led public engagement (Dawson, 2011). Without wanting to dispute the importance of education and incentives, the problems with this kind of public health are well-known. Predefining or circumscribing what counts as healthy (and what matters to people), neglecting diverse life experiences, discounting the confusing deluge of often conflicting health messages and sources of information, and ignoring the assets and capacities, as well as the social and material constraints, that affect everyday practices, all result in approaches that can alienate people, underestimate complexity, undermine health initiatives, and widen health inequalities.

Co-creating and sustaining conditions for health and well-being require something more than this top-down version of public health. They involve a different understanding of what public can mean. In this we draw on two traditions of thinking about the term (see Rock, 2017; Mahony and Stephenson, 2017 and Mahony et al. 2010). The first, the discursive tradition, suggests that people come together to formally and, in western traditions, rationally debate a matter or issue, and so form a public. The second is less exclusive and stems from what Rock (2017) calls a materialist tradition. Here a public signals a particular collective of people, their relations with each other and with a host of other bodies and matters (from genes to atmospheric processes). In both cases, instead of the public being out there, already constituted and passively waiting to be informed (and in some versions made to conform to a more rational world of the health professional), in this approach publics are actively and collaboratively brought into being. In Box 2, we compare how approaches to antibiotic resistance can alter once publics become matters to constitute rather than assume.

This alternative version of emergent and collective approaches to re-defining, creating and sustaining healthy publics requires considerable time, resources and work. Here, we lay out a number of relevant and interrelated points.

First, health and well-being are seldom achieved alone or in isolation. Being healthy often requires carers, families, friends, professionals and many others. Perhaps less obviously, this health collective is not just about people. From the food we eat to the social relations and environment conditions that get under our skin, health is made through and with all manner of others. Recent conceptual reinvestments in epigenetics (the study of the role of environment in modifying genetic processes) through to Planetary and One Health concerns (the pursuit of combined and inter-related human, animal and environmental health, see Craddock and Hinchliffe, 2015) remind us that healthy publics are *heterogeneous*, marked by an interplay of the molecular, the embodied, the social, cultural, environmental, and global.

This more-than- or post -human (see Cohn and Lynch, 2017) aspect of healthy publics generates challenges of participation and speech. Yet we take it that all actors, including people, genes, animals, geologies, chemicals and so on, are potentially active in the development of a healthy public. Some of these actors are more straightforward than others, of course, but if they are vital constituents of the emerging public then a key task will be to work out how best to sense or register their contributions. Here, the work of sociologists and philosophers on the impediments to speech that are faced by all members of a collective (both human and nonhuman) (Latour, 2013) and the role of expert and experienced intermediaries as spokespeople for nonhumans (Stengers, 2010) provide a key resource for assembling a public. The result can be a careful and hesitant involvement of both people and others in ways that underline that a healthy public is not something that can be produced at will. Healthy publics are bound or obligated to human and nonhuman realities (Stengers, 2005, p 192).

Second, these heterogeneous publics will have their own politics and complexities as they struggle to define which realities matter; and to compose themselves in ways that confound any simple ‘additional’ model of assembly (Mol and Law, 2002). How they manage the mix of personnel, expertise, ideas, data and evidence becomes an issue for experimentation and evaluation. Here, recent interrogations of the creativity and fraught practice associated with doing interdisciplinarity are relevant. As Barry and colleagues have insisted, interdisciplinarity can, at its best, be inventive and novel. It can take the form of “public experiments” (Barry et al. 2008), that challenge norms and speculate on new possibilities. This creative destruction is by no means straightforward and needs to be cognisant of existing commitments to forms of evidence, to institutionalised practices, and the uneven power relations that work to undermine potentially new forms of activity and understanding (Hinchliffe et al., 2014).

As Callard and Fitzgerald chronicle in detail, working across and outside disciplines may be a “fractious” endeavour (Callard and Fitzgerald, 2015, p 7). Far from simply staging a conversation, sharing knowledge and achieving mutual respect, these public experiments will only be fulfilling if they successfully identify, often on a case-by-case basis, how co-operation can work in practice. They coin the term “entangled experiments” (Callard and Fitzgerald, 2015, p 8) to capture this relational knot of activity, and note how, for social science and humanities scholars working with natural scientists in particular, mutuality is often of necessity displaced by a strategic form of subjugation.

Strategies for creating the conditions for healthy publics will need to learn from and add to these honest accounts of working together. But as the issues and power dynamics shift as we work with both other researchers and with non-researchers, who may be significantly more or less ‘powerful’ than academics, then we expect both mutuality and subjugation to be joined by other terms that express the range of possible relational styles that make working together possible, productive and enjoyable. The figure of the diplomat (Stengers, 2005), intent on maintaining relations and dealing with contrasts in order to avoid conflicts, may be salient and has been mobilised in flooding publics (Whatmore and Landstrom, 2011). Knowledge brokering, stripped of its translational or educational overtones, may provide another relational role that needs to be developed (Roman et al., 2016).

Third, healthy publics are clearly conditioned by the ways in which public services and public life are more broadly constituted. In national and global contexts, the nature of the relation between states, people, and health-related knowledge and service provision has shifted radically in the last four decades. The rise of agencies, contractors, partnerships, privatised providers, markets, and quasi-markets within national health provision (Newman and Clarke, 2009; Lewis, 1992) is matched by the emergence of new forms of private innovation and philanthropy in global health programmes (Mcgoey, 2015; Craddock, 2017). In science, ‘knowledge economies’ with a premium set on commercial and proprietorial innovation may reduce the public nature of biomedical and public health research; while the move to shareware and open data may conversely have instilled new possibilities for healthy publics to form. The extent to which, and what kinds of, healthy publics are possible given a particular set of public and private service and knowledge provisions is a key and pressing question within administrations, locally, nationally, and internationally. It is also a matter for historical and comparative analyses which can shape how we understand the cultural contexts of health and well-being, and enable the conditions for healthy publics to emerge.

Fourth, healthy publics are dynamic: it takes on-going work to facilitate a thriving collective from the diversity of interests and identities in social settings that may be described as fragmented, complex, global, information rich, and characterised by widening inequalities. Of central concern here are the roles that various media, technologies, data, and materials can play in creating the conditions for a public to emerge, and for sustaining or otherwise suppressing any such public.

The role of technological mediation of biomedicine, social media and the internet in allowing otherwise dispersed communities living with orphan diseases to make a disease public is a good example (Tempini, 2015). The distribution of new kinds of environmental monitoring and citizen science projects that allow air quality to be measured in real time provides another illustration of how healthy publics can emerge (Gabrys, 2016). Again, issues concerning the forms and qualities of acceptable evidence become key matters for sustaining these healthy publics, as do questions of power and the role of commercial and other organisations who may, for example, have interests in sustaining decidedly unhealthy social and material relations. The citizen sensing project’s (Gabrys et al., 2016) reflexive approach to the ways in which the data in polluted communities are generated as well as curated or ‘creatured,’ is a clear example of the need to develop research designs (and funding schemes) that can work across a full research cycle in for a public to be assembled and sustained.

Fifth, there is a need to understand how and in what ways health becomes a problem around which a public can form. Often the very knowledge and technologies that make a healthy public possible (such as expertise, monitoring devices, data analysis, medical imaging and medical science), can also alienate or distance people from health issues. A public that is ‘at once intimately affected by the issues’ (the air we breathe, the condition we live with), can find itself ‘at a remove from’ the knowledge and platforms that are in place to publicise those issues; intimate connection to, as well as potential alienation from, public matters characterises many current health issues and cultures (Marres, 2012, p 31). An example would include the bio-medicalization of mental health, where everyday stresses are reconstituted as potentially socially stigmatising diagnoses and mood disorders are modelled according to notions of chemical imbalances that are, at best, unsubstantiated and contested and, at worst, reinforce psycho-pharmaceutical interventions that carry risks of adverse effects and low rates of compliance (Healy, 1987, 1997, 2004, 2006; Moncrieff, 2007; Lacasse and Leo 2015). Similarly the offshore production of randomised control trials for Pre-Exposure Prophylactic interventions (PrEP) for HIV produced a form of knowledge that was considered by patient groups to be unethical and unrepresentative of the intimacies of day-to-day exposure risks and the practicalities of medicine use (Michael and Rosengarten, 2013).

The medicalization and institutionalisation of health coupled with disenfranchisement and disaffection with expertise makes healthy publics a problem space requiring fresh approaches and experimentation. We need to assess the extent to which new kinds of participation in health and well-being (including telecare, personalised medicine and genetic testing as well as ethical consumption, animal care and environmental monitoring and management) can constitute sustainable forms of healthy publics; or whether they simply add to the stresses and strains of sharing responsibilities for health without the powers to make a difference (Martin, 1995).

Finally, this sense of healthy publics as a problem space, where the relationship to matters of health and well-being is felt to be ambiguous or at least far from settled, has implications for health policy. It should be clear, we hope, that a healthy public is not generated through a top-down imposition of what it means to be healthy. Indeed, healthy publics involve a redistribution of expertise, drawing in those who may have previously been excluded or silenced in health debates and controversies, despite their experiences and understanding of cultures and environments of health. Similarly, we doubt the extent to which this problem space can be solved purely through behavioural manipulation of choice architectures.

The problem as we see it is not one of alignment of ‘the public’ with ‘the experts,’ but rather the articulation or joining together of a public. From this perspective, meaningful and on-going engagement is key to allowing publics to generate questions and possible resolutions that are informed by their collective expertise and experiences. Healthy publics are *not* populist or simply bottom-up approaches to health and well-being. They require and are made in concert with the collaboration and crafts of cutting-edge medical sciences, social sciences and humanities, and with all manner of nonhuman ‘things that force thought’ (Stengers, 2010), in order to form collective ways of knowing and appropriate forms of evidence. The key point here is the will to continually work across and outside previous areas of expertise and practice; to co-identify questions and approaches that are relevant to all those affected by the issues; and to generate appropriate forms of evidence that can allow this health and wellbeing knowledge to circulate, gain traction, and contribute towards effective health-care practices and policies. This we would suggest is a description of a truly transdisciplinary and healthy public endeavour.

## Enabling healthy publics through engaged research

Just as deficit-based approaches to public health presume a target population made up of individual people, so academic approaches can tend to assume a pre-constituted public for research. In contrast, healthy publics must be co-generated through research partnerships and practices which focus on relational, rather than individual, dimensions of health, and which seek to alter the nature and quality of those relations (White, 2017). These may range, as we have suggested, from the social relations that characterise a community, to the ecological and material relations that make health possible to the systemic inequalities, institutional and other structural determinants that shape how opportunities for health and well-being are unevenly distributed (Dutta, 2010). The corresponding research approach is one which embraces, rather than ignores or seeks to ‘allow for’, the complexities of the places, networks and environments in which people live, and looks to create opportunities to generate and foster new kinds of healthy relations.

Of course, engaged research is not a new concept or practice. Indeed, before the advent of laboratory and hospital-based Western medicine in the nineteenth century (Pickstone, 2001), it was not uncommon for doctors to engage with, and immerse themselves in the work and lives of, people around them as a means of generating shared forms of knowledge about health, well-being, and disease (Ramazzini, 1780). In more recent health research in the UK, which is dominated by the need to provide quantitative evidence generated by controlled trials, descriptions of how patients and carers will be involved in proposed research, or a detailed justification as to why their involvement in shaping the research is not possible, is a pre-requisite for most funding streams. This requirement for patient and public involvement in funded health research sits alongside the creation of organisations to support public involvement and engagement.^2^

Even so, much of the research regarding behavioural interventions in health and well-being takes an instrumental or consumerist approach to public engagement (Purtell et al., 2011). A group or segment of the population may be consulted for example about eating well or exercising more, or about policies designed to communicate pointed health messages, but this ‘consult and target’ approach rarely leads to a healthy public being generated through the co-design of an intervention or through the adoption of a relational approach to understanding the cultures within which behaviours are expressed. Although systematic reviews have highlighted the potential of participatory research approaches to improve health outcomes and address health inequalities (Agency for Healthcare Research and Quality, 2004; Hicks S et al., 2012; Bagnall et al., 2016), far less has been written about how the dynamics of doing engaged research can help to create the conditions from which healthy publics might emerge.

Our conceptualisation of engaged research for healthy publics is one that incorporates multiple voices and recognises multiple forms of knowledge and experiences. Engaging from the outset as well as throughout and beyond the research life-cycle, this approach acknowledges that healthy publics are not necessarily bounded by organisations or geography, that they are not constituted simply by their health condition or caring role, and cannot be reduced to mere representation. Rather engaged research facilitates the emergence of healthy publics through sharing experiences and information, provides opportunities to challenge dominant systems of knowledge, and creates possibilities for new practices and care pathways.

A particular feature of this approach is that it involves researchers engaging people who have traditionally been marginalised or excluded from health-related research, as well as incorporating the non-human dimensions of health, such as the health of ecosystems and trans-species health. Since such endeavours allow better understandings of how health and wellbeing are framed within different constituencies and how socio-cultural and environmental relations affect health, they can enable the co-creation of culturally sensitive and more appropriate responses to health and well-being (See Box 3).

Transformative engaged research necessitates building relationships that acknowledge and understand people’s lived experiences. Rather than start with individuals as targets for health messages, such as ‘eat well, exercise more’, engaged research generates recognition of, and has respect for, what people identify as barriers to their health, and these processes of engagement help to create the conditions for trusting and mutually respectful relations to form (Durie et al. 2017, Durie et al. n.d.). Crafting the conditions for these relations to support engaged research requires a negotiation of the research questions and methods, as well as expectations as to what can be delivered, in order to ensure that the research is of benefit to non-academic partners.

However, pursuing meaningfully engaged research requires an awareness of often profound differences in perspectives both within and between academic disciplines, and between service users, carers and practitioners (Rose, 2014). And benefits are unlikely to be shared evenly. For example, monitoring of antibacterial resistance or environmental pollution may well be essential in building a knowledge base for relevant healthy publics, but these activities (including aspects of citizen science) may be difficult to fund and generate little in the way of esteem and publications for university-based researchers.

The capacity to co-create and conduct research in this manner requires institutional structures and processes to support engaged research, as well as clear and transparent roles and responsibilities for the conduct and delivery of research. Given the dynamics of engagement - their ebb and flow - it also requires consideration of a ‘follow-on’ phase, once the research project has been completed, for people to reflect on whether there are additional research questions to address together in the future.

Rejecting approaches that define or segment populations in terms of their ‘problems’ (such as health conditions, health-related behaviours or degraded environments), or in terms of the manner in which they merely represent people with pre-identified health conditions, engaged research regards publics as self-organising and ‘emergent’ in response to the processes of engagement themselves.^3^ Evidencing and evaluating the varied impacts of this approach requires capturing the dynamics of engagement and the resultant partnerships, as well as the outcomes. Only in this way, can we generate greater understanding of how transdisciplinary engaged research can itself help to create the conditions for healthy publics.

As we have already argued, engaged research is likely to involve public and entangled experimentation (Barry et al. 2008; Callard and Fitzgerald, 2015), generating new alliances and encounters in ways that do not shy away from the fractious politics of building, repairing, and sustaining relationships. Heeding Callard and Fitzgerald’s (2015) call to avoid easy recourse to languages of mutuality and sharing, we need to ensure that engaged research involves experimentation in and an evaluation of the kinds of relationships that are productive and sustainable. Similarly, there is a need to investigate how new forms of evidence can be made public, gain traction, and effect change.

## Conclusions

The proposition that health and well-being require publics may not sound particularly new. But this seemingly innocent inversion of public health contains far-reaching challenges to existing research and policy approaches. Healthy publics are evidently ‘in the making,’ and as such are fragmentary and fragile compositions, conditioned by geographical and historical specificities, and requiring new and often demanding ways of working. They take culture seriously, and so require humanities and social researchers to trace the resources for thinking and acting differently. In doing so, scholars should be allowed and encouraged to use a full range of methods and styles of evidence, subject of course to the established standards of evaluation in these areas.

But more than a remote or solely scholarly affair, healthy publics assemble a range of lay expertise and lived experiences, alongside biomedical and social science as well as humanities, to reconfigure a problem or generate collective outcomes. Ageing well across the life-course, for example, requires not just investment in biomedical remedies for later life cognitive impairment, but also recognition of the relational, cultural and environmental–that is, non-pharmacological–factors that enable people to cope with life crises and transitions. Care facilities and practices and local services need to be attendant to a range of cognitive and functional abilities, and to a host of other areas in building healthy public spaces. The challenges of antibiotic resistance require attendance to the multiple rationalities of current medicinal uses in order for us to create and sustain effective medicines for the future. The value of performative approaches to allow people to explore alternative futures is important to the health outcomes of younger age groups, as well to those who might want to explore other possible pathways to healthy outcomes. In such instances, culture is not seen as a barrier to the implementation of better health; rather, culture offers us evidence, resources and possibilities for building and sustaining healthy publics.

Finally, as we have been keen to emphasise, any attempt to adopt a ‘fifth’ or ‘cultural wave’ of public health requires a shift not only in what we think publics are, but also how they are engaged in research. Healthy publics are collectives that take seriously the social and environmental relations that make health and well-being possible. They do so through a process that is neither top-down nor bottom up, but compositional, enabling the development of alliances where questions and approaches to improving health and well-being are co-created.

## Box 1 Midlife crises and transitions: cultural perspectives

In Western discourses, the life cycle has traditionally been divided into stages that are loosely framed in terms of the perceived biological and psychological changes that occur at significant points of transition, such as puberty and the menopause. For example, while accounts of ageing in men have been dominated by the notion of the ‘midlife crisis,’ which is understood as a psychological struggle for personal identity in the face of death, experiences of midlife transitions in women have traditionally been linked simplistically to the menopause or ‘the change.’

As a number of historians, anthropologists and literary scholars have argued, definitions and experiences of life stages and the transitions that connect them are shaped by cultural expectations and social relations as much as they are by alterations to our bodies (Lock, 1993; Gullette, 1997, 2004; Mintz, 2015). Thus, carefully contextualised studies of perceptions and representations of ageing in the past and present demonstrate how clichéd narratives of individual crises at midlife persistently fail to acknowledge the diverse determinants of health and the complexity of experiences during middle age or the impact of midlife challenges on the health and well-being of families.

Analysis of historical records from the National Marriage Guidance Council and the Tavistock Clinic–which include details of the impact of the breakdown of relationships on partners, but only rarely on their children–as well as contemporary fictional and cinematic sources, indicates how different narratives of midlife carry different psychological, emotional and political meanings in different cultural settings (Gullette, 2004); and how transitions through midlife are recounted, experienced and regulated in diverse ways. Richer understandings of ageing and crisis–and their consequences for health across an individual life course and for the well-being of others–can be revealed by situating such sources within the context of changing conceptions and expectations of the family; fluctuating patterns of marriage, work, and divorce; and competing interpretations of autonomy, self-fulfilment, and responsibility (Beck and Beck-Gernsheim, 1995; Lewis, 2001).

Challenging dominant–often stereotypical and discriminatory–narratives of the life course and enabling people to age well through midlife requires attention to new sources and interdisciplinary methods and novel forms of engaged research that reveal the cultural, relational and environmental contexts of crisis and transition.

## Box 2 Rational medicine use or an antibiotic public?

From their first uses in the 1940s and 1950s, antibiotics have been enveloped in debates about inappropriate and rational use. Initially, the role of pharmaceutical companies in encouraging prescription and marketing fixed dose combinations became a target for pharmacologists concerned with encouraging rational use based on evidence from randomised control trials as opposed to sometimes spurious testimonials (Podolsky, 2014). More recently, fears of a post-antibiotic era brought about by widespread antibiotic resistance, itself judged to be a result of over and inappropriate medicine uses, have sparked another push for rational therapeutics in human and animal health (Davies, 2013).

And yet, if rational use is defined only in terms of narrowly framed biomedical science, or through often contestable clinical trials, we end up with a classic problem of wrongly framing prescribers, patients and others as misguided. The results can be ineffective. Telling a general practitioner or primary health care doctor that it is irrational to prescribe antibiotics to a patient with non-specific symptoms might miss the effects of; the lengthy time delays should they opt for diagnostics; the litigious fear of letting a condition go untreated; the time and cost pressures on their practice; or a patient’s need to receive medical affirmation of an otherwise complex condition. Similarly, telling a farmer that using antibiotic-laced feed to improve the health of their livestock is irrational may miss the cultural economy of just-in-time pressures of production, and the need to fulfil a contract and make a living (Hinchliffe et al., 2016). In both cases, there is a tendency to ignore the context or situated nature of treatment practices and, more than incidentally, an underplaying of the ways in which private benefits are valorised in practice at the expense of public, collective or commons costs.

An alternative approach starts from the proposition that rather than ignoring these practicalities or labelling them as irrational, they become the matters around which a public can form. A collective may be built for example that takes prescription data, or a participatory ‘one health’ model for delineating the flows of antimicrobial residues within a rural environment, as a means to gather a diverse public who can act together on the issues at hand. An antibiotic public is thus mediated and co-generated rather than addressed or corrected.

## Box 3 Transitioning to independent living: a relational approach

Young people transitioning from foster care and care homes to independent living are recognised as a high-risk group for behavioural and emotional issues and poor health and educational outcomes (Schofield and Simmons, 2009). Research has mostly focused on developing interventions targeting individual outcomes such as educational attainment or substance abuse, rather than supporting care-leavers in forming new relations that could positively affect their health.

Working with the regional children-in-care team who oversee the transition to independent living, care-leavers and drama practitioners, researchers developed a participatory research approach to understand whether performance-based methods and principles could be used to engage care-leavers in developing new relationships and forming new communities as they transitioned out of formal care. The project used the creation of a theatrical performance and performative activities to enable the co-creation of safe space within with the participants would reimagine their sense of self and their community. These spaces allowed participants to ‘perform’ the creation of new relations, and of imagined or problematized future life scenarios, activities and performances which in turn were used to engage other care-leavers.

This dynamic, relational approach to supporting the transition to independent living started with care-leavers’ narratives of themselves and their peers, and used performance-based methods to engage young people, to develop new relations and communities, and to foster a healthier public in terms of social relations and health outcomes.^4^

## Notes

1 For further information on engaged research approaches, see: http://participationcompass.org/article/index/method; https://www.nice.org.uk/about/nice-communities/public-involvement/patient-and-public-involvement-policy; http://www.ids.ac.uk/team/participation, Accessed 14 June 2017.

2 See for example: www.invo.org.uk; https://www.publicengagement.ac.uk, Accessed 25 Sep 2017.

3 ‘When is Citizen participation transformative?’ http://www.open.edu/openlearn/society/politics-policy-people/participation-now/when-citizen-participation-transformative, Accessed 4 July 2017.

4 See for example: https://connected-communities.org/index.php/project/community-what-do-you-mean-an-investigation-into-how-differing-understandings-of-the-term-community-shapes-care-leavers-move-to-independence/, Accessed 25 Sep 2017.
